# Diversity of Termites Used in Poultry Feed in Burkina Faso

**DOI:** 10.3390/insects16070687

**Published:** 2025-06-30

**Authors:** Aïchatou Nadia Christelle Dao, Fernand Sankara, Mouhamadou Moustapha Ndiaye, Abdoulaye Baïla Ndiaye, Salimata Pousga, Irénée Somda, Marc Kenis

**Affiliations:** 1 Institut du Développement Rural (IDR), Université Nazi Boni (UNB), Bobo-Dioulasso 01 BP 1091, Burkina Faso; ferdisank2005@yahoo.fr (F.S.); pousgasalimata@yahoo.fr (S.P.); ireneesomda@yahoo.fr (I.S.); 2 Laboratoire de Zoologie des Invertébrés Terrestres, Institut Fondamental d’Afrique Noire Cheikh Anta Diop (IFAN-Ch. A. Diop), Université Cheikh Anta Diop (UCAD), Dakar BP 206, Senegal; mouhamadoumoustapha6.ndiaye@ucad.edu.sn (M.M.N.); abdoulayeb.ndiaye@ucad.edu.sn (A.B.N.); 3 CABI, 1 Rue des Grillons, 2800 Delemont, Switzerland; m.kenis@cabi.org

**Keywords:** local knowledge, termites, morphological identifications, Burkina Faso

## Abstract

The aim of the study was to assess the diversity of termites used in poultry feed in Burkina Faso. Termite samples were collected in eight of the thirteen regions of the country by poultry farmers, then conserved in 70° alcohol. The criteria used by poultry farmers for identifying the termite were also characterised and discussed with farmers in a village where the use of termites as poultry feed is well developed. Morphological identifications were carried out in the laboratory. Twenty species were identified in two families, six subfamilies, and thirteen genera. In the Heterotermitidae family, a single species belonging to the Coptotermitinae subfamily was identified. The rest belonged to the family Termitidae and the subfamilies Amitermitinae, Microcerotermitinae, Macrotermitinae, Nasutitermitinae, and Cubitermitinae. Three species, *Microcerotermes fuscotibialis*, *Megagnathotermes notandus*, and *Isognathotermes fungifaber*, were found for the first time in Burkina Faso. The largest number of species (eleven) was collected in the Cascades region. Poultry farmers are able to recognise eight genera of termites by the shape, size, and colour of the termites; by the termite mounds; and often by the location of the nest. These results may facilitate the promotion of the use of termites as poultry feed in Burkina Faso and West Africa.

## Simple Summary

There are various species of termites in West Africa. Some of them are used as protein sources in poultry feed by rural poultry farmers. The diversity of species depends on the climatic zone. As a result, the species actually used by poultry farmers are not well known. To improve knowledge of the termite species used by poultry farmers in Burkina Faso, information was collected from poultry farmers on their local knowledge of termites and samples of termites were taken. They were analysed in the laboratory on the basis of morphological criteria. The results will help to improve poultry farmers’ knowledge of termites and facilitate their use in West Africa and Burkina Faso.

## 1. Introduction

Termites and their mounds are used in many parts of the world for human food and animal feed [1,2]. Their traditional use in animal feed, particularly poultry feed, has been recently investigated in West Africa [3,4,5,6,7]. Termites which are used as a supplement for scavenging poultry are collected in the wild by breaking their mounds or by trapping them in inverted containers filled with organic matter and placed on the soil near termite nests [5,6]. Termites are rich in proteins, lipids, minerals, and essential amino acids [8,9,10,11,12,13]. Many authors have mentioned the benefits of these insects on poultry production performance [4,14,15]. By using termites, poultry farmers can reduce the high cost of animal proteins (fish or meat meal), which are particularly expensive in Sahelian countries such as Burkina Faso [4]. Using termites in poultry feed enables small-scale farmers in rural areas to provide their poultry with quality protein at a lower cost. In Burkina Faso, 78% of poultry farmers use termites in their birds’ feed at least occasionally [3]. The termite species used in poultry feed in the different agro-climatic zones of Burkina Faso belong mostly to the genera *Macrotermes*, *Trinervitermes*, and *Odontotermes* [6,16]. However, other genera are sometimes used, and many species are not yet listed in the literature. The limited knowledge of the termite species by rural poultry farmers is one of the reasons why this available source of protein is being abandoned [3]. Few studies have been conducted on the termite species used by rural poultry farmers. Most of the documents available provide punctual information on the types of termites used or describe the effect of a species on poultry. It is, therefore, necessary to be able to clearly identify the termites used to supplement poultry diets in Burkina Faso to draw up an exhaustive list of species for the country. This list will be reviewed and expanded as termite studies progress. This study was conducted to (i) identify the different termite species used in poultry farming in Burkina Faso, (ii) determine the regional distribution of the different species, and (iii) evaluate the diversity of criteria used by poultry farmers to identify termite species.

## 2. Materials and Methods

**Figure 1:**
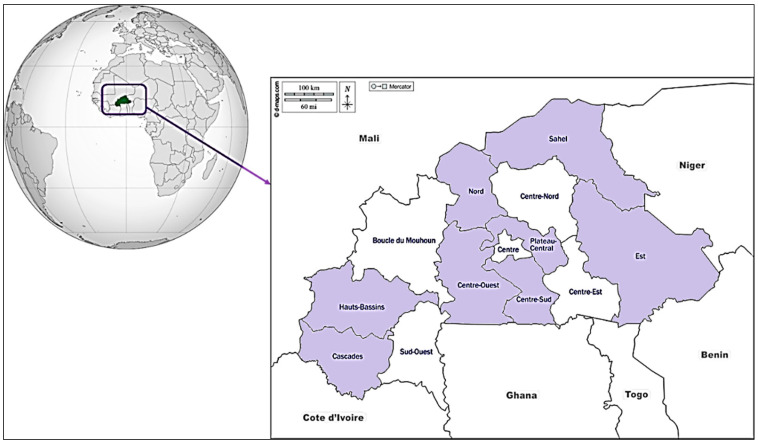
Regions surveyed for termites used as poultry feed in Burkina Faso; 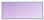 Regions covered by the surveys.

Study area: The termite specimens were collected by rural poultry farmers during surveys in the regions of Centre Ouest, Plateau Central, Nord, Est, Sahel, Centre Sud, Cascades, and Hauts-Bassins (Figure 1). They represent the three eco-climatic zones of Burkina Faso and cover more than 60% of the country as follows: the Sahelian zone (Sahel and part of Nord); the North Sudanian zone, or Sudano-Sahelian zone (Centre Ouest, Plateau Central, Est, and part of Nord and Centre Sud); and the South Sudanian zone (Hauts-Bassins, Cascades, and part of Centre Sud).

Sampling technique: In 2015, 1100 farmers from all investigated regions were asked to collect termites using their traditional methods (see details of the surveys in [3]). Some farmers collected broken termite mounds containing termites and others trapped them with overturned containers filled with humid organic matter. A description of the different techniques is provided by [6]. The termites were sampled from the termite mound residues and from the traps using flexible forceps and placed in vials containing 70° ethanol.

Insect identification: Identifications of the termites were carried out in the laboratory of Zoologie des Invertébrés Terrestres (ZIT) of the Institut Fondamental d’Afrique Noire Cheikh Anta Diop (IFAN—Ch. A. Diop) of the Université Cheikh Anta Diop (UCAD) in Dakar, Sénégal. The termites were identified by the authors, following [17,18,19,20,21,22,23,24,25,26,27,28]. Identifications were based on the morphological characters of the soldiers, in particular through morphometric measurements (length of head, width of head, length of left mandible—the thickness of the head for Nasutitermitinae). A Leica M80 stereomicroscope equipped with an IC 80 HD camera connected to a computer was used for observations, followed by the digitalization of the specimens. The images obtained using Leica Application Suite Version 4.9 (LAS V4.9) software were then examined, measured, and compared with those of reference specimens from the laboratory’s collection acquired under the same conditions. The main morphometric characters used for identification were observed on the head, the mandibles, and the posterior tibia of the soldiers and/or workers [25]. More specifically, measurements were made of the length of the head, in dorsal view, from the occiput to the base of the labrum. The width of the head corresponds to its greatest width in the dorsal view. The length of the left mandible in dorsal view was measured from the apophysis to the tip.

Exceptionally for the Cubitermitinae, dissections were carried out on the workers, in addition to the measurements taken on the soldiers, in order to examine the structure of their enteric valves. Dissections were carried out in 70° ethanol under a stereomicroscope. The segment of the digestive tract containing the enteric valve was removed and then emptied of its contents using an ultrasound bath. To study the chitinous structures, the enteric valve was first cut lengthwise, then dehydrated with 90° ethanol. Finally, it was placed between a slide and a coverslip using Euparal gasoline as the mounting liquid.

Identification of termites by farmers: An additional study was conducted in 2019 in the village of Siniéna in the Cascades region, where earlier surveys had shown that the use of termites as feed is particularly developed. Ten model poultry farmers recognised by the local authorities as being termite users in all seasons were chosen for individual interviews to establish the main criteria enabling them to differentiate between the types of termites in the field. They were then asked to specify the vernacular names of the different ‘species’ they knew. These names were collected during the sampling and confirmed later by all the poultry farmers.

## 3. Results

### 3.1. Termite Species Used in Poultry Feed in Burkina Faso

**Figure 2:**
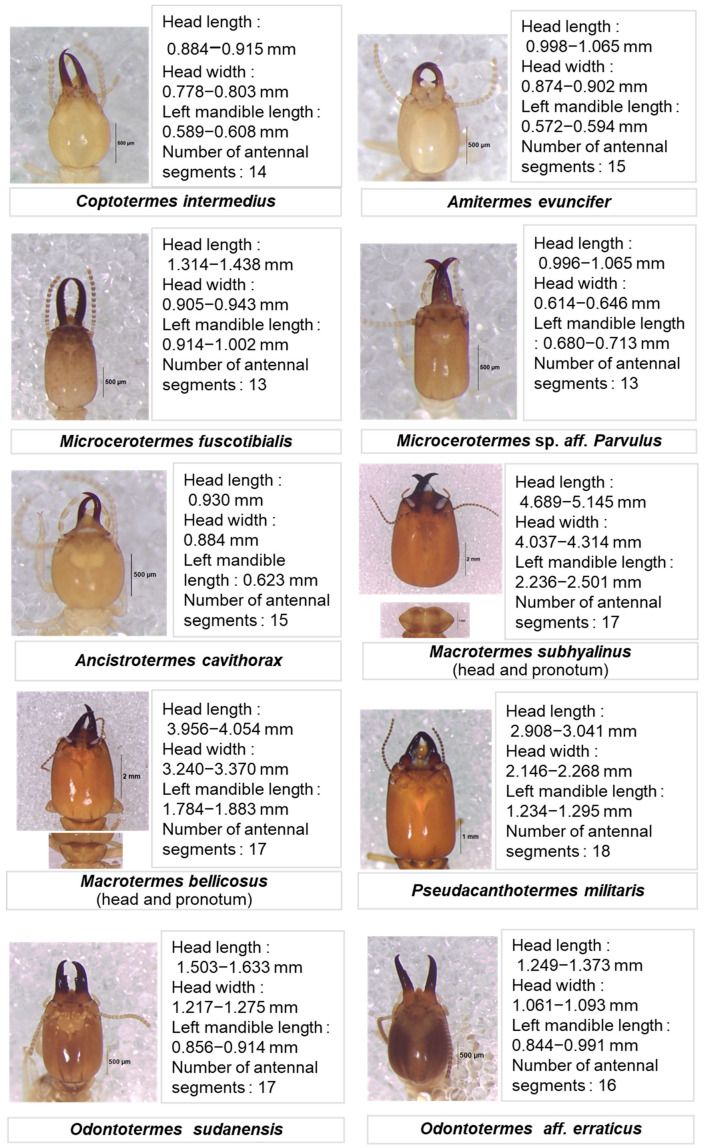

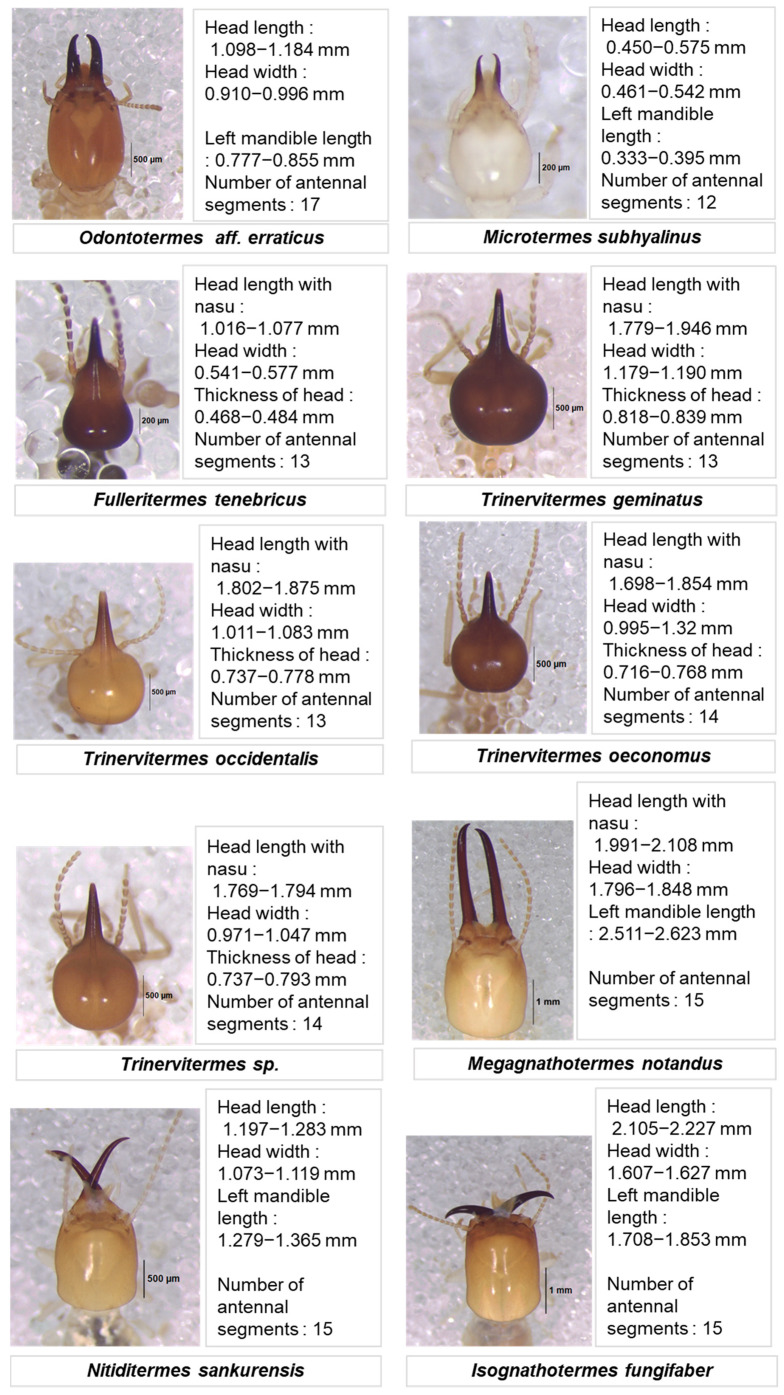
Termite soldiers’ heads used in poultry feed in Burkina Faso.

**Figure 3:**
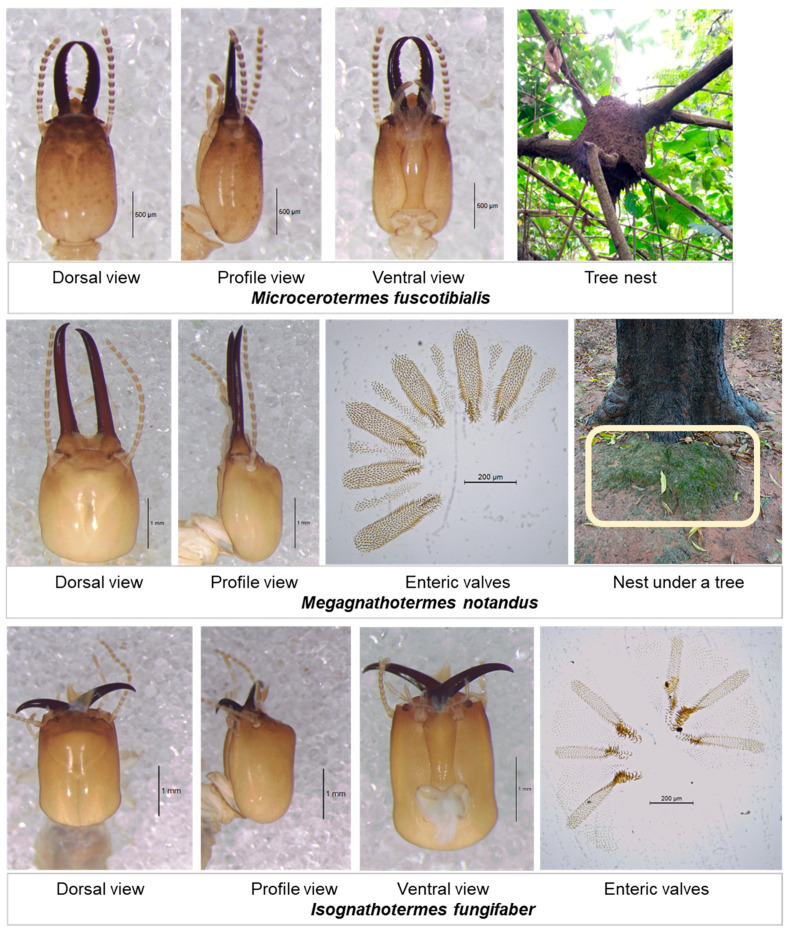
Soldiers’ heads and enteric valves of workers and/or nests of termite species mentioned for the first time in Burkina Faso.

The identification of the termites collected during the surveys revealed twenty species in two families, five subfamilies, and thirteen genera (Table 1). Figure 2 shows illustrations of the soldiers’ heads of the termite species identified with measurements and the number of antennal articles. Three species represent new records for Burkina Faso, the Microcerotermitinae *Microcerotermes fuscotibialis*, the Cubitermitinae *Megagnathotermes notandus*, and *Isognathotermes fungifaber* (Figure 3).

Two genera of termites were found in all the study regions, *Macrotermes* spp. and *Odontotermes* spp. Three species were found in seven of the eight regions as follows: *Macrotermes bellicosus* (Smeathman), *M. subhyalinus* (Rambur), and *Nitiditermes sankurensis* (Wasmann). The greatest number of termite species collected was observed in the Cascades region (16/20 species), followed by Hauts-Bassins (11/20 species), Centre Ouest (7/20 species), and Est (7/20 species). The lowest number of species was collected in the Sahel (2/20 species) (Table 2).

### 3.2. Identification of Termites by Farmers

**Figure 4:**
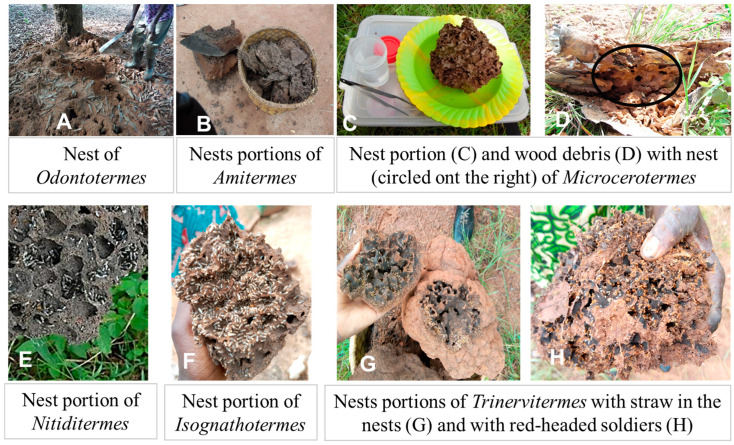
Illustrations of termite nests and parts of nests by genus. The circle shows nest of *Microcerotermes* on wood debris.

Several termites’ genera are used for poultry feed by farmers of the Village of Siniena. The criteria that help poultry farmers recognise termites in the field were identified through observations and discussions during termite harvests. These criteria are based essentially on the size, shape, colour, and location of the termite mound and the colour of the termites (Figure 4 and Table 3). *Macrotermes* is a termite genus that farmers easily identify through their large size and the height of their termite mounds. Farmers are able to distinguish the two species, saying that *M. subyhalinus* have darker head capsules and make mounds that are more rounded than in *M. bellicosus*. The two *Macrotermes* spp. are given different local names (Table 3). *Odontotermes* spp. are small termites that poultry farmers can recognise by their activities on the ground, the whitish colour of their thorax and abdomen, their reddish head, and also by their termite mound, which has holes where termites can be seen building the mounds in the rainy season (Figure 4A).

*Amitermes* spp. build dark-coloured termite mounds on stems of palmyra palm (Borassus akeassii) (Figure 4B). These termites are generally found in decomposing plant debris, but especially on palmyra palm, hence the name “palmyra palm termites” used by poultry farmers. All the galleries built by these termites are black in colour and give off a strong, characteristic odour.

*Microcerotermes* spp. are often called “daba termites” in reference to the hypogeous nature of certain nests (Figure 4C). The “daba” (traditional hand hoe) comes across a nest that is being harvested at the same time as the farmer is clearing the land. This species is very rare and very nutritious, according to the farmers. As a result, it is given to chicks as a priority. *Microcerotermes* spp. can also have arboreal nests found in wet habitats, such as the banks of watercourses. They can be found in trees or on decomposing plant debris (Figure 4D).

Nests of the genera *Nitiditermes* and *Isognathotermes* are dark, brownish to blackish (Figure 4E,F), and contain termite species with a black abdomen. Soldiers have a reddish head and workers a whitish head. The termite mound may have one or more hats, or none at all. Poultry farmers make no distinction between these species. *Megagnathotermes* is another genus of Cubitermitinae that is usually confused with the two other genera. Farmers give the same local name (Tchegnelhon) to these three genera with dark termite mounds.

*Trinervitermes* spp. mounds are identified by growers thanks to the straw bales stored in the mound by the termites (Figure 4G) and also thanks to the presence of Andropogon grass in the area around the mound. The soldiers’ heads are often dark. Termites of the genus *Trinervitermes* can also be recognised by the presence of small holes in their termite mounds. If a sound is made near the termite mound, the soldiers, with their reddish heads (Figure 4H), start to emerge through the often clearly visible holes.

## 4. Discussion

The identification of termites has revealed twenty species belonging to two families, the Heterotermitidae and the Termitidae. In the Heterotermitidae family, only one sub-family has been identified, the Coptotermitinae. In the Termitidae family, species belonging to the subfamilies Amitermitinae, Microcerotermitinae, Macrotermitinae, Nasutitermitinae, and Cubitermitinae were found. Of the twenty species identified, the following three are new to Burkina Faso: *Microcerotermes fuscotibialis*, *Megagnathotermes notandus*, and *Isognathotermes fungifaber*. These three species were sampled in the Cascades region where more humid climatic conditions favour the establishment of humivorous termites [29]. The species *Isognathotermes fungifaber* and *Nitiditermes sankurensis*, both humivorous termites, belonged to the genus *Cubitermes* until a recent revision [27]. The species mentioned as *Cubitermes* sp. in previous studies on termites as commonly used as livestock feed in West Africa [5,6,16] was most likely *N. sankurensis*. Several other species found during this study were only recently reported from Burkina Faso, i.e., *Odontotermes sudanensis*, *Pseudacanthotermes militaris*, and *Fulleritermes tenebricus* [30] and *Coptotermes intermedius, Amitermes evuncifer*, and *Odontotermes* aff. *erraticus* [31]. Furthermore, an unidentified species of *Trinervitermes* found during our surveys could be a new undescribed species.

Some species-level identifications should be considered with caution, in particular in the genera *Odontotermes* and *Trinervitermes*. During identifications, intraspecific variations were observed in the genus *Odontotermes* with two types of soldiers of different colours. These variations may be due to the edaphic conditions of the environment. Intraspecific variations may also be linked to the origin and developmental stage of individuals [25]. There are also strong similarities between the different species of *Odontotermes*, making them difficult to identify. The genus *Odontotermes* in Africa should be revised, including using molecular methods [30]. The identification of *Trinervitermes* spp. is also challenging, based on the measurements of the soldiers, even though variations in the shape of antennal articles can help [23]. For this genus as well, molecular approaches should be used.

Of all the species identified, the genera *Macrotermes* and *Odontotermes* were the only ones found in all regions, which can be explained by the strong adaptation of the termites of the subfamily Macrotermitinae to the ecological and edaphic conditions of the environment. Indeed, in terms of organisation, it is the best-organised species that adapts to different conditions [32]. The unequal distribution of termite species in different regions shows that the presence of termites in a region can be effectively influenced by the environment and climatic factors [33]. The high number and diversity of termite species identified in regions located in the southern Sudanian zone (Cascades and Hauts—Bassins) and the low number of species identified in the Sahelian zone (Nord) had been noted by [29] along the climatic gradient in Burkina Faso. The Sahelian zone is the most arid zone in Burkina Faso, while the Sudanian zone is the most humid. Evapotranspiration is also very high in the Sahelian zone [34]. These climatic factors may explain the difference in termite diversity observed in these different zones. The presence of *Macrotermes* spp. and *Odontotermes* spp. species in all the regions studied can also be explained by a potential bias in our surveys caused by the fact that poultry farmers particularly target these genera using the trapping technique with inverted containers [6]. These termites have a very high nutritional quality and can be used to improve poultry feed at a lower cost [4,7,15]. The two other genera commonly collected for poultry feed in Burkina Faso and West Africa are *Trinervitermes* and *Cubitermes* s.l. (probably *Nitiditermes*, see [27], which are collected by destroying nests, usually in the rainy season) [5,6]. Previous studies had already shown that they are not commonly harvested in the Sahelian region, possibly because the soil is very dry for most of the year in this region [6].

The criteria used by poultry farmers to recognise the different genera of termites are essentially based on the location, size, shape, and colour of the nests, as well as the morphology of the individuals. These criteria can be quite practical but not totally reliable, as several factors can influence criteria such as the shape and colour of the nests. This explains why termite genera in the Cubitermitinae subfamily share the same local name. There are even stronger similarities between species, making it difficult to discriminate between them [25]. However, the recognition criteria work well for certain genera of termites and enable wise poultry farmers to diversify the termites harvested for poultry feed.

## 5. Conclusions

The large number of termite species used in poultry feed in Burkina Faso demonstrates the availability of this resource to poultry farmers. The main genera used by farmers, i.e., *Macrotermes*, *Odontotermes*, and *Trinervitermes*, are found in nearly all regions, and they represent an opportunity for traditional poultry farmers to provide their poultry with a quality protein supplement. The existence of the concept of termite species among poultry farmers, who have given them names based on certain criteria, may facilitate the dissemination of knowledge about termites.

## Figures and Tables

**Table 1:** List of termite species used in poultry feed in Burkina Faso, identified by family and subfamily.

Family	Subfamily	Genus	Species	Bibliography
Heterotermitidae	Coptotermitinae	*Coptotermes*	*Coptotermes intermedius* (Silvestri, 1912)	[27]
Termitidae	Amitermitinae	*Amitermes*	*Amitermes evuncifer* (Silvestri, 1912)	[24]
	Microcerotermitinae	*Microcerotermes*	*Microcerotermes fuscotibialis* (Sjöstedt, 1896) *	[25,27]
			*Microcerotermes* aff. *parvulus*	
	Macrotermitinae	*Ancistrotermes*	*Ancistrotermes cavithorax* (Sjöstedt, 1897)	[18]
		*Macrotermes*	*Macrotermes subhyalynus* (Rambur, 1842)	[19]
			*Macrotermes bellicosus* (Smeathman, 1781)	
		*Odontotermes*	*Odontotermes sudanensis* (Sjösted, 1924)	[21]
			*Odontotermes* aff. *erraticus* (form 1 and 2) (Grassé, 1944)	[22,25]
		*Pseudacanthotermes*	*Pseudacanthotermes militaris* (Hagen, 1858)	[20]
		*Microtermes*	*Microtermes subhyalinus* (Silvestri, 1914)	[21]
	Nasutitermitinae	*Fulleritermes*	*Fulleritermes tenebricus* (Silvestri, 1914)	[23]
		*Trinervitermes*	Trinervitermes *geminatus* (Wasmann, 1897)	
			*Trinervitermes occidentalis* (Sjöstedt, 1904)	
			*Trinervitermes oeconomus* (Trägardh, 1904)	
			Trinervitermes sp.	
	Cubitermitinae	*Isognanthotermes*	*Isognathotermes fungifaber* (Sjöstedt, 1896) *	[26,28]
		*Megagnathotermes*	*Megagnathotermes notandus* (Silvestri 1973) *	[27,28]
		*Nitiditermes*	*Nitiditermes sankurensis* (Wasmann, 1911)	[26,28]

* New record for Burkina Faso.

**Table 2:** Distribution of termite species according to the region of collection.

Species	Centre Ouest	Plateau Central	Nord	Est	Sahel	Centre Sud	Cascades	Hauts-Bassins
*Coptotermes intermedius* (Silvestri, 1912)							X	
*Amitermes evuncifer* (Silvestri, 1912)				X			X	
*Microcerotermes fuscotibialis* (Sjöstedt, 1896)							X	
*Microcerotermes* sp. aff. *parvulus*							X	
*Isognathotermes fungifaber* (Sjöstedt, 1896)							X	
*Megagnathotermes notandus* (Silvestri)							X	
*Nitiditermes sankurensis* (Wasmann, 1911)	X	X	X	X		X	X	X
*Ancistrotermes cavithorax* (Sjöstedt, 1897)				X				
*Macrotermes subhyalynus* (Rambur, 1842)	X	X	X	X		X	X	X
*Macrotermes bellicosus* (Smeathman, 1781)	X	X	X		X	X	X	X
*Odontotermes sudanensis* (Sjösted, 1924)	X	X				X	X	X
*Odontotermes* aff. *erraticus* 1 (Grassé, 1944)							X	X
*Odontotermes* aff. *erraticus* 2 (Grassé, 1944)	X		X	X	X		X	
*Pseudacanthotermes militaris* (Hagen, 1858)							X	X
*Microtermes subhyalinus* (Silvestri, 1914)							X	X
*Fulleritermes tenebricus* (Silvestri,1914)								X
*Trinervitermes geminatus* (Wasmann, 1897)				X				X
*Trinervitermes occidentalis* (Sjöstedt, 1904)	X		X				X	
*Trinervitermes oeconomus* (Trägardh, 1904)	X						X	X
*Trinervitermes* sp.	X	X		X		X		X
**20**	**8**	**5**	**5**	**7**	**2**	**5**	**16**	**11**

**Table 3:** Some criteria used by poultry farmers to recognise termite genera.

	Recognition Criteria														
	Termite Mounds											Termites			
	Size		Shape				Colour	Location				Colour			Local Name and Genus or Species
	Large	Small	Round	In Cathedral	Presence of Hole	With Hat (s)	Dark	In the Palmyra Palm	Other Tree	On the Ground	In the Ground	Whitish	Yellowish to Reddish	Dark	
1	X		X							X				X	Hiwallo *M. subhyalinus*
2	X			X						X			X		Tchilahon *M. bellicosus*
3	X		X		X					X		X			Gnemissenhon (small termites) *Odontotermes*
4		X				X	X			X				X	Tcheyelhon: *N. sankurensis*
5							X	X						X	Gnongnorhon (Termites of palmyra palm): *Amitermes*
6		X			X					X			X		Sahoré: *Trinervitermes*
7		X								X			X		Tiena (near andropogons) *Trinervitermes*
8		X					X		X			X			Gneplahon (termites blancs) *Microcerotermes*
9		X									X		X		Tiegnehon (termites of daba) *Microcerotermes*
10											X		X		Bikognenhon *Pseudacanthotermes*
11											X	X			Halalanga *Microtermes*

## Data Availability

The original contributions presented in this study are included in the article. Further inquiries can be directed to the corresponding author.
